# Direct Sinus Lift With Delayed Implant Placement Using a Bovine-Derived Xenograft and Collagen Membrane: A Case Report

**DOI:** 10.7759/cureus.98934

**Published:** 2025-12-10

**Authors:** Sangeeta Rai, Vasudha Gupta, Ashutosh Dixit, Shambhavi Kumar

**Affiliations:** 1 Dentistry, All India Institute of Medical Sciences, Rishikesh, Rishikesh, IND; 2 Periodontology, Subharti Dental College, Meerut, IND

**Keywords:** bovine xenograft, collagen membrane, delayed implant placement, posterior maxilla, risk factors, sinus lift

## Abstract

Rehabilitation of the posterior maxilla with dental implants can be challenging because of reduced bone height, poor bone quality, and sinus pneumatization. This report describes the treatment of a severely atrophic posterior maxilla with a residual ridge height of 2.43 mm using a lateral window (direct) sinus lift. A bovine-derived xenograft was used in conjunction with a resorbable collagen membrane, followed by delayed placement of two implants and prosthetic restoration. A 43-year-old female patient underwent sinus augmentation with deproteinized bovine bone mineral and collagen membrane. After nine months of healing, two Bioline implants (4.2×13 mm) were inserted and later restored with a screw-retained prosthesis. One-year follow-up demonstrated stable marginal bone, healthy peri-implant tissues, and excellent esthetic and functional outcomes. This case highlights the reliability of direct sinus augmentation using xenograft and membrane in sites with extremely limited bone height and also discusses potential risks and limitations of the procedure.

## Introduction

Rehabilitation of the atrophic posterior maxilla with dental implants remains a clinical challenge due to pneumatization of the maxillary sinus and progressive loss of alveolar bone height following tooth extraction. These anatomical limitations often prevent conventional implant placement and necessitate sinus augmentation to recreate sufficient vertical dimension for implant stability [1,2]. Over the years, several sinus-elevation techniques have been introduced, broadly categorized into lateral window approaches and transcrestal (osteotome or graftless) elevation methods. The lateral window technique allows direct visualization and substantial augmentation, making it suitable for severely resorbed ridges, while the transcrestal approach is considered less invasive and is often preferred when moderate residual bone height is available [3,4].

More recent innovations have focused on reducing surgical morbidity by using graftless sinus-elevation protocols, in which implant placement itself provides tenting support for the Schneiderian membrane. These techniques rely on the natural capacity of the sinus cavity to form new bone through inward migration of osteoprogenitor cells, particularly when membrane integrity is preserved [5,6]. Additionally, platelet concentrates have gained attention for their potential to enhance healing, reduce post-operative complications, and improve early vascularization in minimally invasive sinus-elevation procedures [7].

The choice of sinus augmentation method must also consider sinus anatomy, membrane thickness, sinus width, and the quality of residual bone, as these factors influence both the predictability and bone regeneration potential of each technique [8]. When the sinus is narrow, and membrane elevation is achievable without excessive tension, graftless or minimally grafted approaches may offer high success rates while avoiding complications associated with xenograft or allograft materials [9]. Considering these anatomical and biological principles, the selected technique in the present case aimed to minimize invasiveness while supporting predictable implant integration through a controlled sinus-elevation protocol consistent with current evidence-based recommendations [10].

## Case presentation

A 43-year-old female presented to the department of periodontology and implant dentistry with the chief complaint of difficulty chewing in the upper left posterior region for the past several months. The patient also expressed an interest in fixed prosthetic rehabilitation to replace her missing teeth in that area. Her medical history was noncontributory, with no known systemic diseases, allergies, or history of smoking, alcohol use, or sinus pathology. She was not on any long-term medications, and her vital signs were within normal limits. Her dental history revealed the extraction of teeth #26 and #27 approximately one year earlier due to caries. No previous grafting procedures had been performed at the site.

Extraoral examination showed no facial asymmetry, swelling, or tenderness. There was no evidence of sinus-related symptoms, such as nasal obstruction, discharge, or facial pressure. Intraoral examination revealed missing teeth #26 and #27, with healed edentulous mucosa. The ridge appeared narrow in the vertical dimension in the premolar-molar region. The adjacent tooth (#25) exhibited abrasion but no caries or periodontal pockets. The edentulous site presented with adequate keratinized mucosa and no signs of active inflammation. Palpation revealed a firm but vertically deficient alveolar ridge.

**Figure 1:**
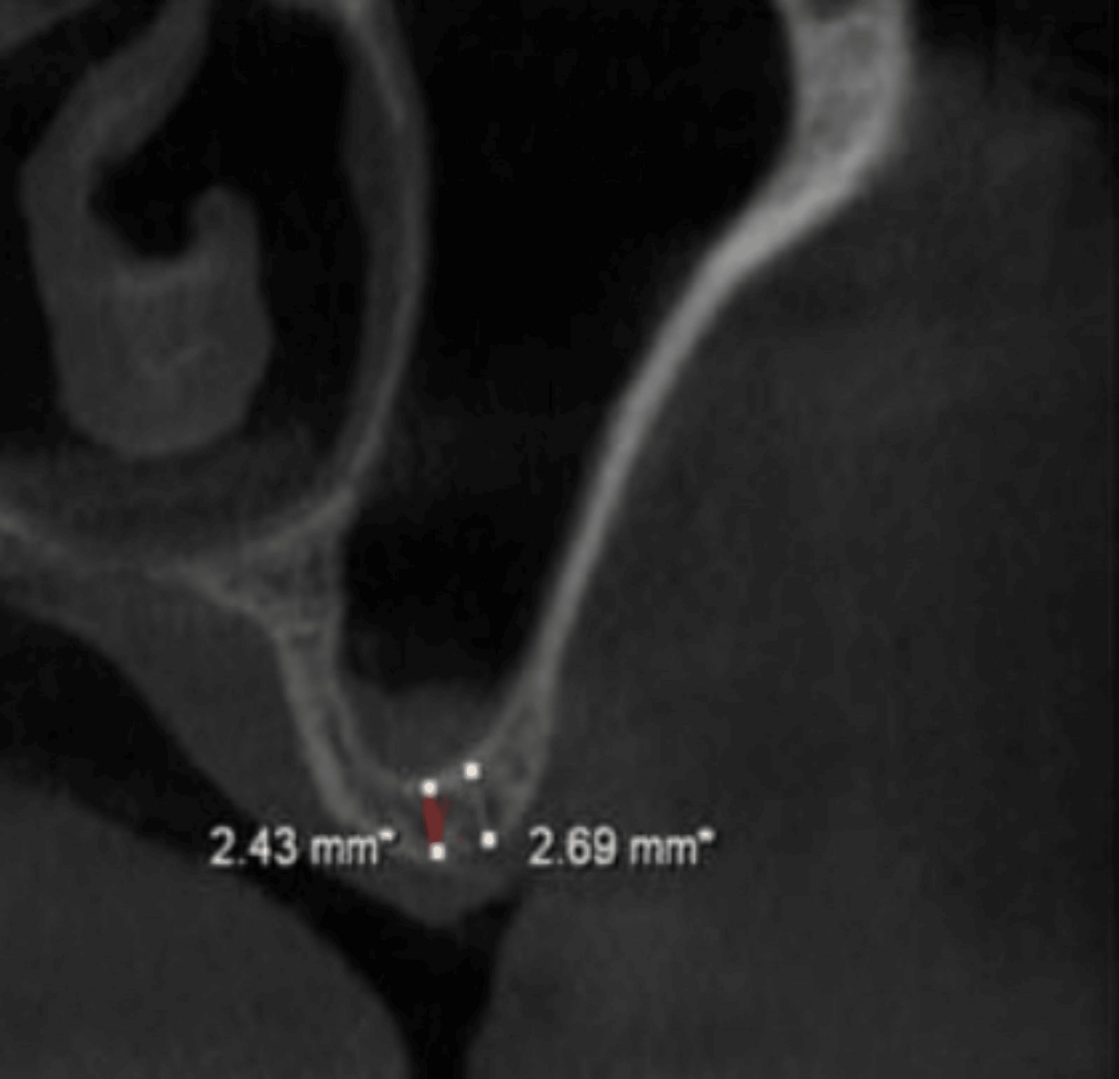
Preoperative CBCT depicting 2.43 mm residual ridge height under the sinus floor. CBCT: cone-beam computed tomography

A periapical radiograph showed reduced vertical height beneath the left maxillary sinus. To obtain a more accurate anatomical assessment, cone-beam computed tomography (CBCT) was performed. The CBCT scan demonstrated a pneumatized left maxillary sinus with a residual alveolar bone height of 2.43 mm in the region corresponding to teeth #26 and #27 (Figure 1). The lateral sinus wall was of moderate thickness, and the sinus membrane appeared intact with no evidence of mucosal thickening, septa, or sinus floor irregularities. Based on the available bone height, a direct (lateral window) sinus augmentation was deemed necessary to permit subsequent implant placement. Delayed implant placement was selected due to the severely limited native bone height and the need for sufficient graft maturation.

A variety of graft materials have been used for sinus augmentation, including autogenous bone, allografts, xenografts, and alloplastic substitutes, each with distinct strengths and limitations. Autogenous bone offers osteogenic capacity but is associated with higher resorption and donor-site morbidity. Allografts eliminate harvesting complications but show variable remodeling potential. Alloplasts provide biocompatibility but often lack long-term dimensional stability. Bovine-derived xenografts, in contrast, demonstrate excellent volume stability, slow resorption, and well-established clinical success, making them particularly suitable in cases with severely reduced residual ridge height. Given the patient’s minimal native bone height (2.43 mm), the need for delayed implant placement, and the desire to avoid additional morbidity, bovine-derived xenograft with a collagen membrane was selected as the most predictable and biologically stable option.

**Figure 2:**
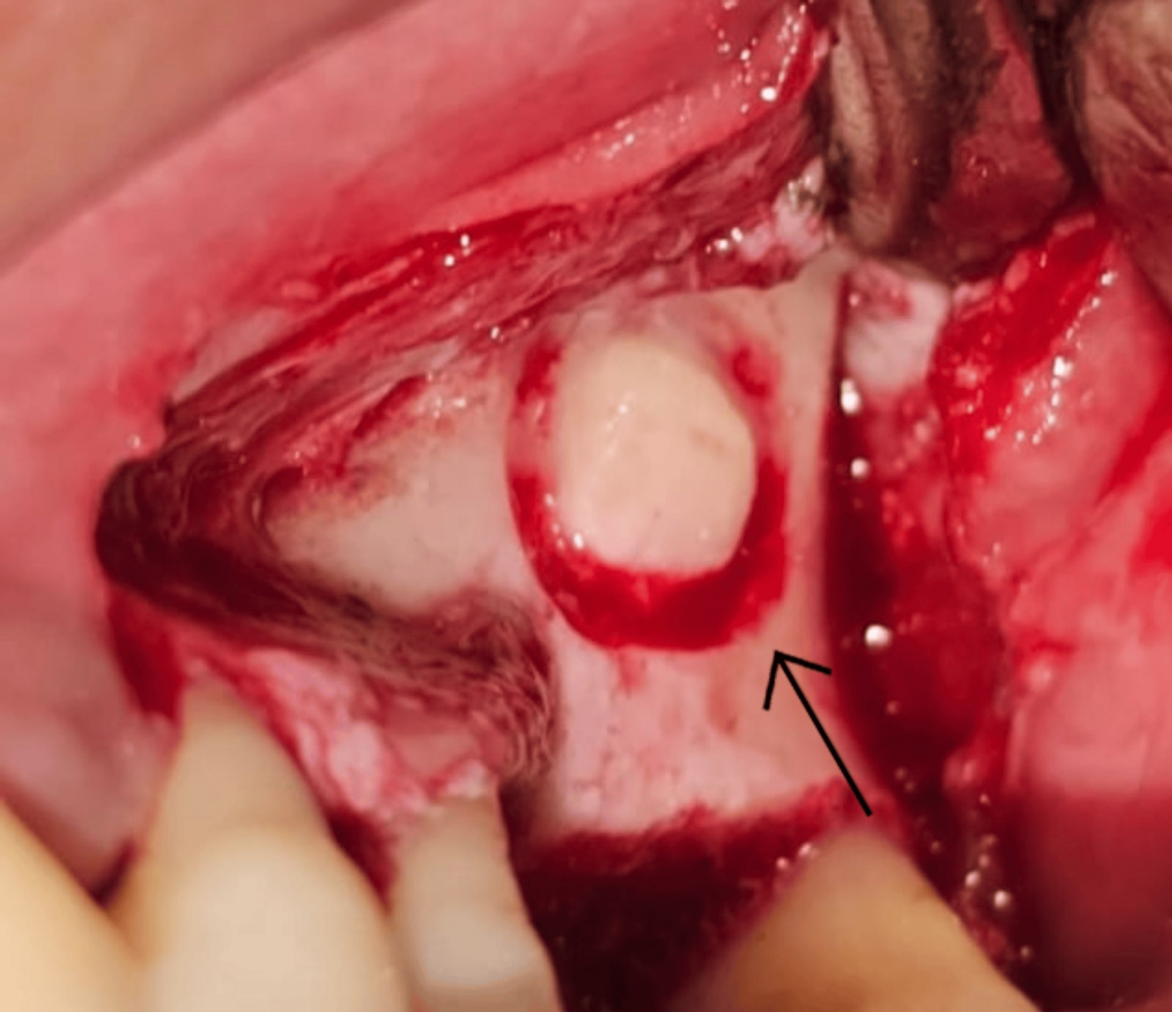
Reflection of the full-thickness flap showing lateral wall exposure and window preparation. The arrow indicates the inferior border of the bony window.

**Figure 3:**
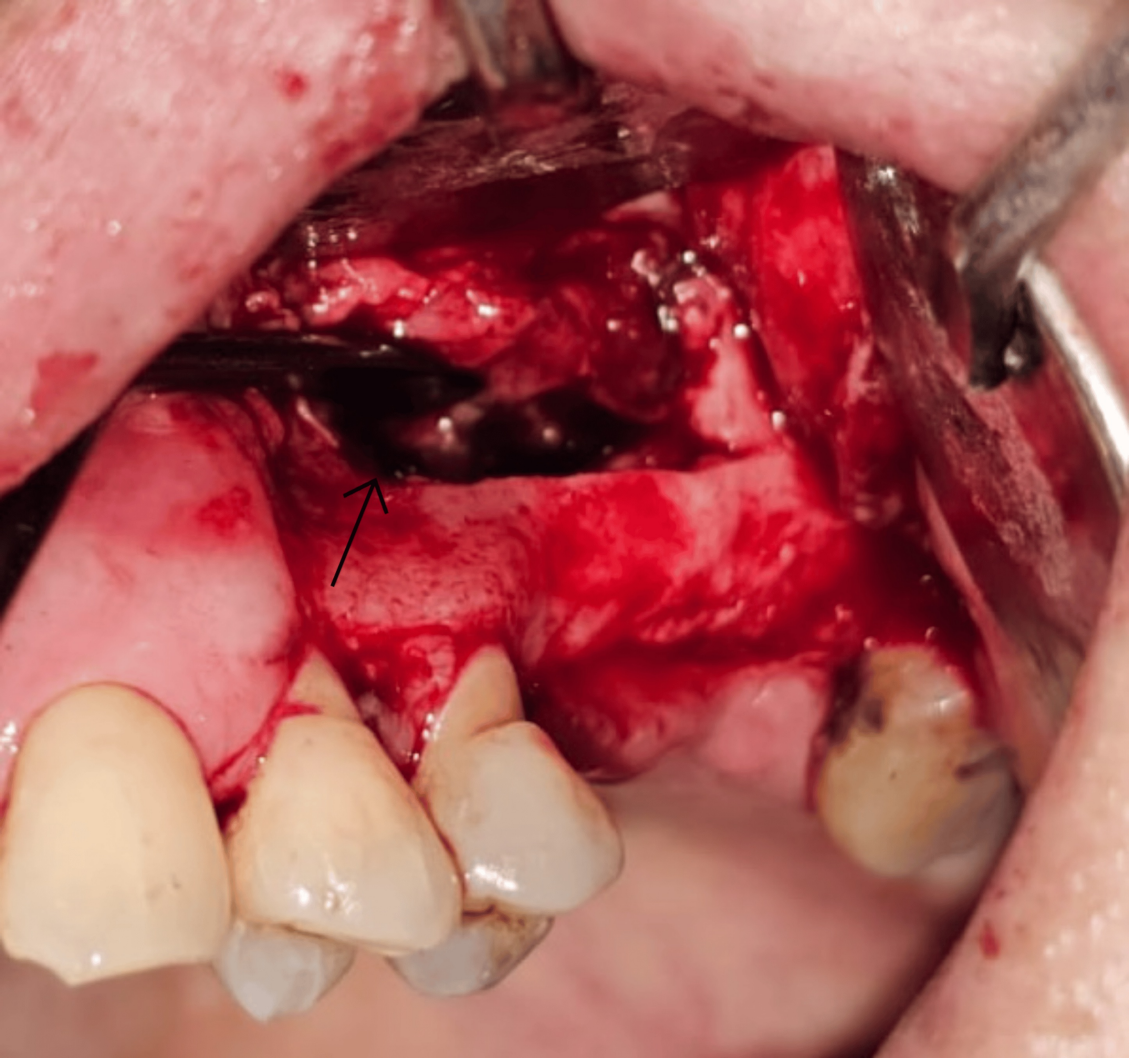
Elevation of the Schneiderian membrane without perforation. The arrow indicates the lifted Schneiderian membrane with the help of a sinus lifting instrument.

Prior to surgery, the surgical site was disinfected using 0.12% chlorhexidine gluconate mouthwash for 1 min. After administration of local anesthesia (2% lignocaine with 1:80,000 adrenaline), a mid-crestal incision and an anterior vertical release were made. A full-thickness trapezoidal flap was reflected to expose the lateral wall of the sinus (Figure 2). Using a round bur on a low-speed straight handpiece under continuous saline irrigation, a rectangular window approximately 12×8 mm was created in the lateral wall. The antral mucosa was then carefully elevated, and the prepared antrostomy was subsequently infracted, like a trap door, and used as the superior border of the sinus compartment, leaving it attached to the underlying Schneiderian membrane, and being careful not to create perforation. The Schneiderian membrane was visualized and carefully elevated till the medial aspect of the sinus, to ensure sufficient space for placement of graft material (Figure 3). The specific tools used are the GDC Sinus Lifting Instruments, such as IMPSL1, IMPSL2, IMPSL3, etc. The sequence of using the instruments is as follows: (1) initial elevation: start by using the initial sinus lift curettes (e.g., IMPSL1, IMPSL2). Begin separating the Schneiderian membrane from the underlying bone with a rotating motion. (2) deeper separation: use more acutely angled curettes (e.g., IMPSL3, IMPSL4) to lift the membrane further into the sinus cavity, ensuring the tip remains in contact with the bone. (3) final elevation: use the final sinus lift curettes (e.g., IMPSL5, IMPSL6, IMPSL8, IMPSL9) with their distinct ends to elevate the membrane from the anterior and posterior aspects. The blunt edges are designed for gentle lifting.

**Figure 4:**
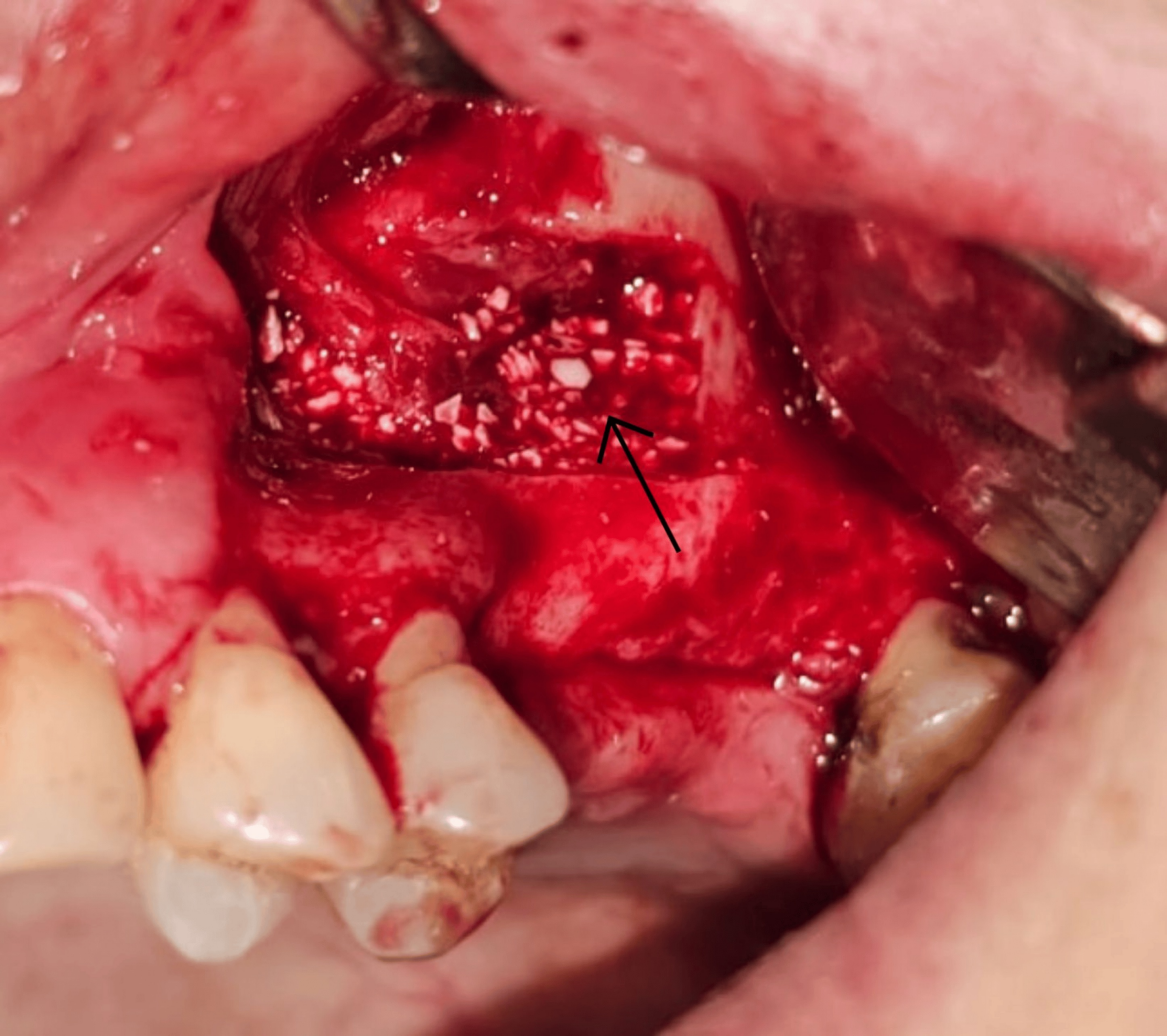
Placement of bovine xenograft material within the elevated sinus cavity. The arrow indicates the placement of the bone graft within the elevated sinus cavity.

**Figure 5:**
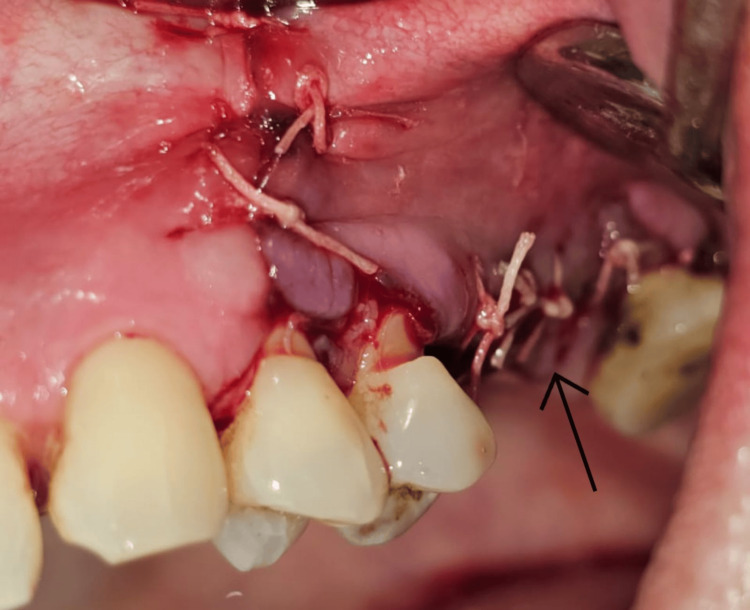
Primary closure achieved with resorbable sutures. The arrow indicates the primary closure of the site with the help of 4-0 polypropylene sutures.

The created cavity was filled incrementally with deproteinized bovine bone mineral (Bio-Oss; Wolhusen, Switzerland: Geistlich Pharma) (Figure 4). A resorbable collagen membrane (Bio-Gide, Wolhusen, Switzerland: Geistlich Pharma) was positioned over the lateral window to seal the grafted area. The flap was repositioned and sutured with 4-0 polypropylene sutures (Ethicon Prolene; Raritan, NJ: Ethicon Inc.) (Figure 5).

**Figure 6:**
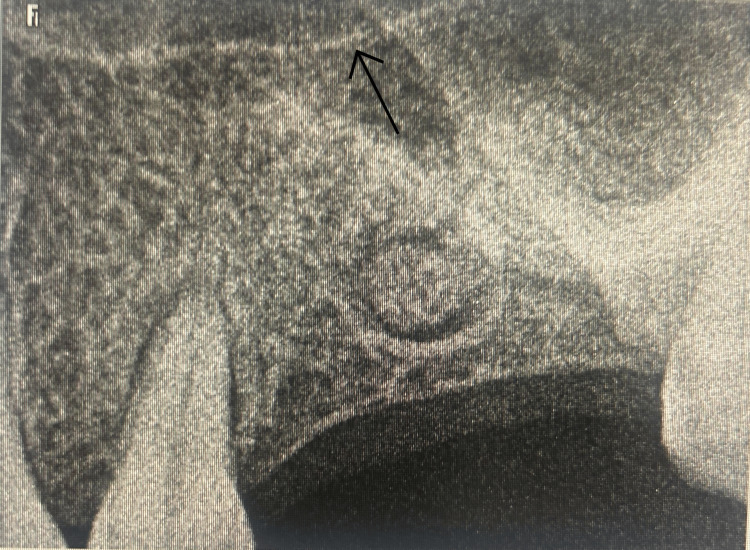
Post-healing IOPA demonstrating augmented bone height. The arrow indicates the lifted sinus membrane. IOPA: intraoral periapical radiograph

Post-operatively, the patient was prescribed amoxicillin 500 mg three times daily for seven days, ibuprofen 400 mg as needed, and instructed to rinse with 0.12% chlorhexidine gluconate twice daily for two weeks. Additionally, she was prescribed dexamethasone 8 mg twice on the first post-operative day, 4 mg twice on the second post-operative day, and 4 mg once on the third post-operative day. She was cautioned to avoid sneezing, nose blowing, and using straws to prevent sinus pressure. Healing progressed uneventfully without complications, such as sinusitis or infection. At nine months post-surgery, a follow-up intraoral periapical radiograph (IOPA) revealed significant bone formation with a vertical height of approximately 16 mm (Figure 6). The graft material appeared well integrated and continuous with the native sinus floor.

**Figure 7:**
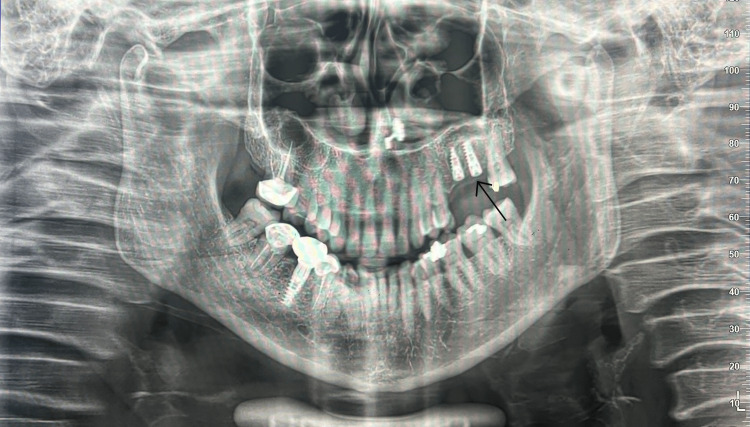
Placement of two Bioline implants (4.2×13 mm). The arrow indicates the placement of two Bioline implants (4.2×13 mm) in the augmented site.

Two Bioline implants (4.2×13 mm) were then placed after nine months, at sites 26 and 27 under local anesthesia. Implant placement was performed freehand, guided by clinical and radiographic assessment of the augmented ridge. The osteotomy was prepared following standard protocol, beginning with a pilot drill and progressing through sequential drills up to the implant diameter (4.2 mm), resulting in an osteotomy prepared to match the implant dimensions. Undersizing was not performed. Both implants achieved primary stability exceeding 35 N cm (Figure 7). Cover screws were attached, and the surgical site was sutured.

**Figure 8:**
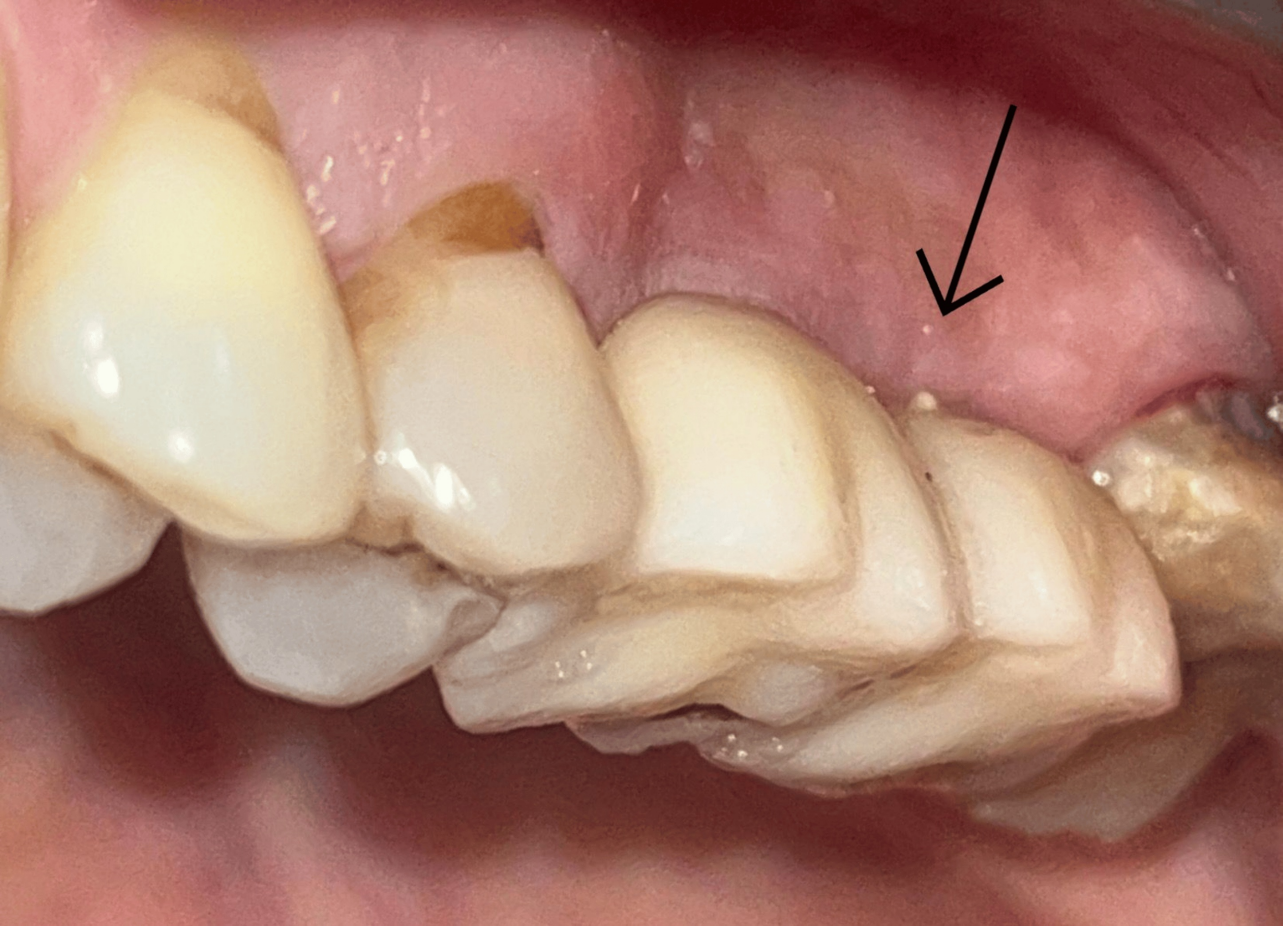
Definitive screw-retained prosthesis in function. The arrow indicates the placement of two screw-retained prosthesis over the implants.

After a three-month osseointegration period, radiographs showed adequate bone-implant contact and no marginal bone loss. Impressions were obtained using an open-tray technique with impression copings. The implants were restored with screw-retained metal-ceramic crowns, ensuring harmonious occlusion and contact points (Figure 8).

**Figure 9:**
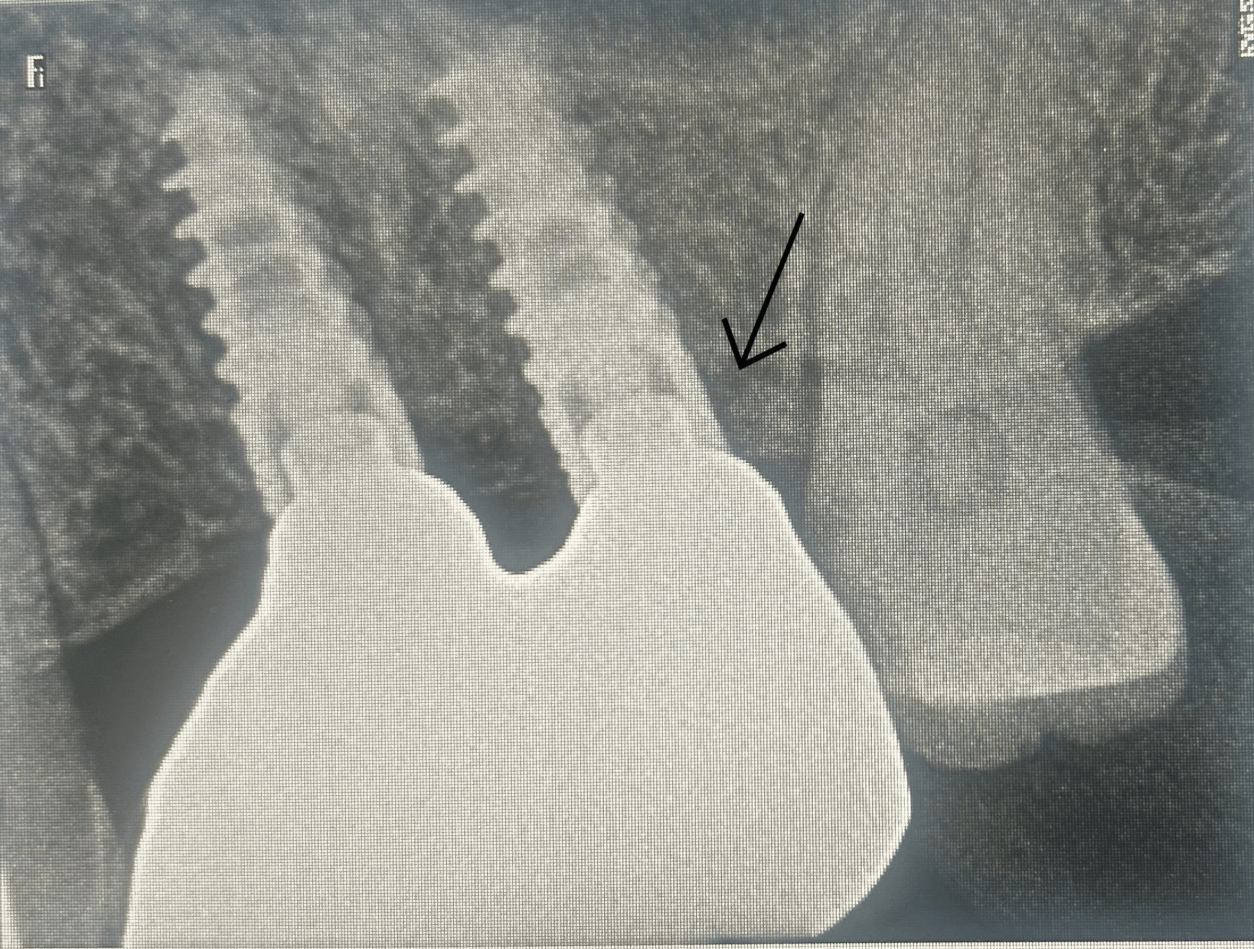
Follow-up IOPA after six months of prosthetic loading. The arrow indicates the marginal bone stability. IOPA: intraoral periapical radiograph

**Figure 10:**
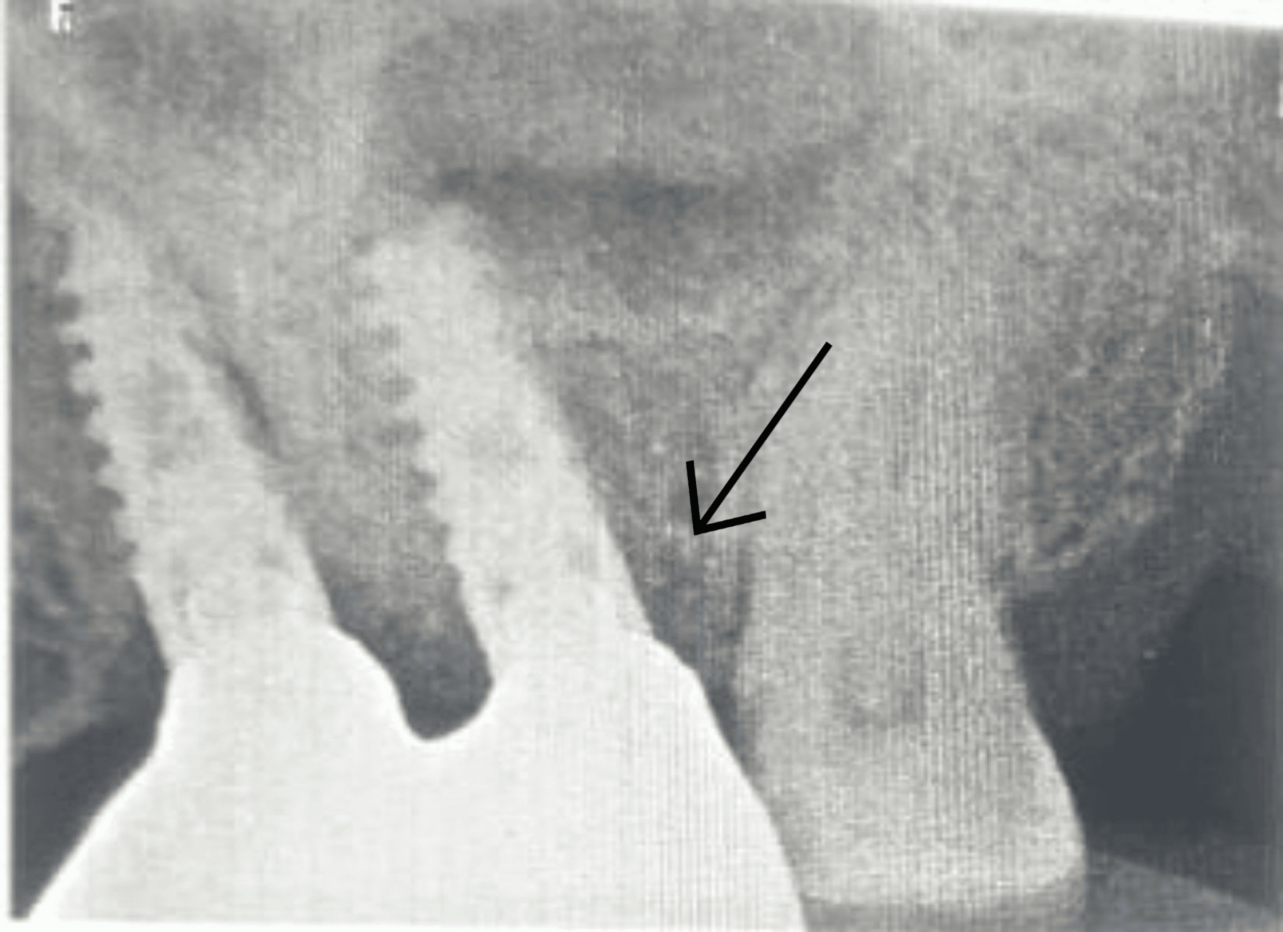
Follow-up IOPA 14 months after prosthetic loading. The arrow indicates the marginal bone stability. IOPA: intraoral periapical radiograph

The patient was monitored at six-month intervals for one year following prosthetic loading. Clinical examination demonstrated healthy peri-implant soft tissues, probing depths below 3 mm, and absence of inflammation. Follow-up radiographs were obtained at six months and one year post-prosthesis delivery (Figures 9, 10).

No bone loss was observed at the six-month follow-up radiograph. However, a minor crestal bone loss was observed at the 14-month follow-up radiograph. The observed bone loss falls within the early physiological remodeling typically expected during the first year of functional loading. This minor crestal change is consistent with the ranges reported in the literature for implants placed in grafted maxillary sinus sites and does not indicate pathological peri-implant bone loss.

The implants remained clinically stable, functional, and free of inflammatory signs throughout follow-up. The evaluation was based on conventional clinical parameters, including soft-tissue health, prosthesis stability, occlusal harmony, absence of discomfort, and patient-reported satisfaction. The patient reported satisfaction in both aesthetic and functional aspects of the prosthesis.

## Discussion

Sinus augmentation remains a critical procedure for implant rehabilitation in the posterior maxilla, particularly in cases with insufficient residual alveolar bone height. A comprehensive understanding of maxillary sinus anatomy is essential in determining the most appropriate augmentation technique. Among anatomical determinants, sinus width plays a crucial clinical role. Narrow sinuses provide a closer approximation of bony walls, facilitating clot stability and predictable bone formation. In contrast, wide sinuses (>12 mm) have been associated with slower bone regeneration and increased membrane tension, thereby requiring more controlled augmentation strategies or staged approaches [10,11].

In recent years, a shift toward minimally invasive, graftless sinus-elevation techniques has been observed, especially when anatomical conditions permit. In a study, it was emphasized that in cases with favorable sinus morphology, particularly narrow sinuses with an intact Schneiderian membrane, the use of platelet concentrates (e.g., concentrated growth factor {CGF}, advanced platelet-rich fibrin plus {A-PRF+}) may enable graftless or minimally grafted sinus elevation while maintaining predictable bone formation and reduced complication rates [12]. Although platelet concentrates were not used in the present case, their emerging role is noteworthy, especially in optimizing healing in graft-reduced approaches.

A two-stage approach was selected in this case based on limited residual bone height, which did not allow for the predictable primary stability required for simultaneous implant placement. Current clinical recommendations generally support a two-stage protocol when residual bone height is <3-4 mm, whereas synchronized implant placement is typically reserved for cases with ≥4-5 mm of native bone or when graftless techniques are feasible [13-15]. Additionally, the unavailability of piezoelectric instrumentation at the time of surgery influenced the decision to rely on conventional rotary techniques, which further justified the staged approach to minimize membrane risk.

Risks and limitations of sinus augmentation

Despite predictable outcomes reported in the literature, sinus augmentation carries inherent risks that must be acknowledged. The most common intraoperative complication is Schneiderian membrane perforation, with reported incidence ranging from 20% to 41% depending on operator experience and sinus anatomy [16,17]. A membrane perforation rate of 41% in 359 lateral-window sinus lifts was reported in a study, which was associated with significantly higher graft-failure rates (11.3% vs. 3.4%) and increased post-operative sinusitis (11.3% vs. 1.4%) in perforated cases [16,18]. Although repaired perforations do not automatically contraindicate implant placement, they remain a significant risk factor.

Graft failure and suboptimal bone regeneration represent additional concerns. Xenografts, such as deproteinized bovine bone mineral (DBBM), although well documented, exhibit slow resorption and delayed remodeling, often leaving residual nonvital particles embedded long-term, which may reduce the proportion of vital bone and affect long-term biomechanical quality [19]. Post-operative complications, including sinusitis, mucosal thickening, and graft migration, are more commonly associated with membrane perforation, graft overpacking, or obstruction of the osteomeatal complex [20].

Long-term complications may include chronic mucosal changes, persistent graft particles, and peri-implant bone loss, even in clinically successful cases. Anatomical variables, such as sinus septa, irregular sinus contours, and thin bony walls, further increase complication risk, as do patient-related factors, including smoking, poor oral hygiene, and systemic conditions. Technique-dependent factors, particularly membrane handling, flap management, and choice of instrumentation, also significantly influence outcomes.

Implications for the present case

Despite a favorable one-year clinical and radiographic outcome, the inherent limitations of sinus augmentation, including membrane-related risks, delayed xenograft remodeling, and potential long-term sinus changes, should be considered. Regular monitoring with periodic imaging remains essential for evaluating graft stability, sinus mucosal health, and peri-implant bone levels. Future protocols may benefit from biologically driven adjuncts, such as platelet concentrates, particularly in cases where sinus anatomy is suitable for graftless elevation.

## Conclusions

A direct sinus lift using a lateral-window approach with deproteinized bovine bone mineral (DBBM) and collagen membrane allowed successful vertical augmentation in the posterior maxilla with residual ridge height of 2.43 mm, enabling delayed placement of two 4.2×13 mm implants and subsequent prosthesis delivery. The technique demonstrated predictable bone volume preservation and satisfactory one-year post-loading clinical outcomes. However, limitations inherent to DBBM, including slow resorption, delayed remodeling, and persistence of residual particles, may reduce the proportion of vital bone and affect long-term bone quality. Additional procedural considerations, such as potential Schneiderian membrane perforation, post-operative sinus complications, and patient-related factors like occlusal overload or habits, highlight the need for meticulous surgical technique and careful follow-up. While this case supports the efficacy of DBBM-based sinus augmentation, future approaches could explore adjunctive strategies - including platelet concentrates or faster-resorbing grafts - to enhance bone vitality and accelerate remodeling, particularly in high-load or esthetic regions.
